# Impact of Substance Use Disorder on Presentation and Short-Term Course of Schizophrenia

**DOI:** 10.1155/2014/280243

**Published:** 2014-04-02

**Authors:** Rudraprosad Chakraborty, Arunima Chatterjee, Suprakash Chaudhury

**Affiliations:** ^1^Berhampore Mental Hospital, Murshidabad, West Bengal, Berhampore 742101, India; ^2^Pravara Institute of Medical Sciences (Deemed University), Rural Medical College, District of Ahmednagar, Loni, Maharashtra 413736, India

## Abstract

The aim of the present study was to compare a cohort of schizophrenia patients with substance use disorder (SUD) with a similar cohort of schizophrenia patients without SUD with regard to sociodemographic variables, clinical variables, psychopathology, anxiety symptoms, depressive symptoms, treatment outcome, and side effect profile of drugs. A total of 143 consecutive inpatients with ICD-10 DCR diagnosis of schizophrenia were included after obtaining informed consent. Patients were evaluated by a semistructured data sheet and Maudsley Addiction Profile. They were then rated by Positive and Negative Symptoms Scale, Calgary Depression Scale, Hamilton Anxiety Rating Scale, and Brief Psychiatric Rating Scale at presentation, three weeks, and six weeks. At three weeks and six weeks, they were also evaluated by UKU Side Effect Rating Scale. Substance abuse was detected in 63.6% schizophrenia patients. Nicotine was the commonest substance followed by cannabis and alcohol. Substance users had longer untreated illness and more depressive symptoms at presentation and six-week follow-up. Dual diagnosis patients had difficulty in abstraction at three and six weeks but not at presentation. Schizophrenia patients with SUD had more depressive symptoms. SUD appeared to mask abstraction difficulties at presentation. Schizophrenia patients with SUD should be carefully assessed for presence of depression.

## 1. Introduction

Substance use disorder (SUD) among schizophrenia patients is an increasingly recognized problem. Prevalence estimates of SUD in schizophrenia patients vary from 17% to 90%, while the rates of DSM IV abuse or dependence range from 28.5% for nicotine, 50.8% for cannabis, and 43.1% to 65% for alcohol [1–3]. There are geographical variations of dual diagnosis prevalence among individuals suffering from schizophrenia. For example, the US prevalence rates highlighted that 47% of subjects with a lifetime diagnosis of schizophrenia or schizophreniform disorder met criteria for an alcohol or a substance use disorder [4]. Somewhat different results were shown in Europe, where the prevalence of dual diagnosis among individuals with schizophrenia ranged from 19 to 35% [5, 6].

There is an ongoing research interest to know the exact relationship between SUD and schizophrenia. There is a suggestion of self-medication; that is, patients take substances to ameliorate depression, anxiety, negative symptoms, or a slowed-down feeling due to extrapyramidal symptoms produced by traditional antipsychotics [7]. It is also suggested that alcohol reduces discomfort of hallucinations [8]; cannabis and stimulants reduce subjective discomfort of antipsychotic side effects as well as negative symptoms [9]; and nicotine reduces cognitive deficits [10]. However, these speculative benefits of SUD are silent about the increase in positive symptoms (cannabis), depression (alcohol), frequently reported medication noncompliance, and worse course of illness in dual diagnosis patients [11, 12]. In addition cannabis use has been associated with an elevated risk of developing psychosis, an earlier age at onset of schizophrenia, and a higher relapse rate after remission of acute psychotic symptoms in the first episode [2, 13]. The comorbidity of SUD and schizophrenia may also be a direct consequence of the underlying neuropathology of schizophrenia, which may contribute to enhanced addiction vulnerability by disrupting the neural substrates that mediate positive reinforcement (reward circuitry dysfunction) [14, 15]. Genetic and gene/environment factors that contribute to schizophrenia may also contribute to addiction [1]. The neural diathesis-stress model suggests that a neurobiological vulnerability interacts with environmental stressors (such as substance use) in vulnerable individuals to precipitate the onset of schizophrenia or relapse of psychosis [16]. The association of SUD with an earlier age at onset of schizophrenia [2] and evidence that patients with schizophrenia experience negative clinical effects, such as relapse, after using small quantities of substances of abuse [17] support this model. The accumulative risk factor hypothesis [18] suggests that people with schizophrenia may have a greater risk of substance use disorder because of the cumulative effects of poor cognitive, social, educational, and vocational functioning as well as poverty, victimization, and exposure to deviant and/or substance-using familial and social environments, all of which are known risk factors for SUD. Overall, the picture appears quite unclear.

Certain unique findings in Indian context can have an impact on the presentation of dual diagnosis patients. For example, prognosis of schizophrenia is better in India compared to many western countries [19]. Nicotine, smoking, and chewing tobacco and cannabis are culturally sanctioned in most parts of India. Similarly, alcohol is part of many traditional tribal cultures particularly in Jharkhand [20]. Even relatively low levels of such comorbid substance use are associated with a poorer outcome, including greater risk of relapse [21, 22]. The potential impact on cognitive function is less clear. It has been claimed that people with schizophrenia and substance use would have better social and cognitive functioning than those who are abstinent [23]. Clinical studies addressing this association have yielded contradictory findings [24, 25]. Hence, it will be interesting to know the interaction of substance use in the psychopathology and course of schizophrenia particularly in this region of India. In view of the foregoing we decided to compare a cohort of schizophrenia patients with SUD with a similar cohort of schizophrenia patients without SUD with regard to sociodemographic variables, clinical variables, psychopathology, anxiety symptoms, depressive symptoms, treatment outcome, and side effect profile of drugs.

## 2. Material and Methods

The study was a longitudinal hospital based study. The project was approved by the institutional ethical committee. Consecutive schizophrenia patients admitted in two large tertiary psychiatric hospitals during the study period formed the sample universe. Patients and their guardian were told about the study in detail and included in the study with their informed consent. Patients were free to withdraw consent during study period and in such circumstances they were dropped from study. Patients who were taken into the study fulfilled the following inclusion and exclusion criteria.

*Inclusion Criteria are*

1. fulfilling ICD 10-diagnostic criteria for research (DCR) [26] of schizophrenia;
2. being drug naïve or drug free for at least four weeks for oral antipsychotics and twelve weeks for depot antipsychotics,
3. being from age group of 18 years–60 years of both sexes.

*Exclusion Criteria are*

1. fulfilling ICD-10-(DCR) [26] Criteria of Substance Induced Psychotic Disorder, organic mental disorder, and personality disorder;
2. being grossly uncooperative for a meaningful assessment (e.g., mental retardation, in acute intoxication or withdrawal, mute).

All the patients with the diagnosis of schizophrenia admitted during the seven-month intake period of study were included in the study after explaining the objectives of the study to the patients and their guardians and obtaining written informed consent. None of the patients refused to participate in the study. The psychiatric diagnosis was initially made by a senior resident and confirmed by the Consultant Psychiatrist. The sociodemographic and clinical details about the participants were collected using a semistructured data sheet developed by us. In addition, each patient was evaluated for their substance use history by using Maudsley Addiction Profile (MAP) [27]. Thereafter each subject was evaluated with the following scales and reevaluated at three weeks and six weeks: Positive and Negative Symptoms Scale (PANSS) [28], Calgary depression scale [29], Hamilton Anxiety Rating Scale (HARS) [30], and Brief Psychiatric Rating Scale (BPRS) [31]. In addition, each participant was evaluated at three and six weeks by UKU Side Effect Rating Scale [32] for any medication-induced side effects. The rating scales were applied by the second author who was blind to the diagnostic group of the patient.

Independent samples* t-*test was performed to examine the differences in continuous variables between schizophrenic persons with SUD (Group 1) and schizophrenic persons without SUD (Group 2). Chi-square test was used in case of discrete variables. To examine whether there was any difference between the rate of symptom resolution between schizophrenic patients with SUD and without SUD, a general linear model repeat measure ANOVA was applied.

## 3. Results

*Sample Characteristics*. A total of 143 patients completed assessment at the end of six weeks. In 108 patients (74.5%) the index episode was the first episode. Average duration of illness was 5.71 (±4.98) years. Average duration of untreated illness was 4.59 (±4.29) years. Average age of onset was 24.38 (±7.48) years. 21 patients (14.5%) had history of suicidal episode in the index episode. Mean age was 29.86 (±8.01) years. There were 134 (92.4%) male and nine female participants. Average education was 9.53 (±4.56) years. 74 persons (51%) were married. 67 (46.2%) were unemployed, 42 (29.0%) were unskilled workers, 16 (11%) were semiskilled workers, and 18 (12.4%) were skilled workers or technicians by profession. 55 (37.9%) persons had their family income less than Rs.2000 per month, 63 (43.4%) had family income between Rs. 2000 and Rs. 5000 per month, and 25 (17.2%) had family income more than Rs. 5000 per month from all sources. Of all the participants, 113 (77.2%) were from extended families and 109 (75.2%) had a rural background.

### 3.1. Prevalence of Substance Abuse

SUD was present in 91 (63.6%) patients, out of whom 86 (60.1%) fulfilled criteria of dependence while 5 patients had harmful use of a substance. The commonest drug of dependence was nicotine (*n* = 82; 57.3%) followed by cannabis (*n* = 25; 17.5%), alcohol (*n* = 16; 11.2%), and solvent (*n* = 1; 0.7%). Dependence on multiple substances was detected in 28 (19.6%) patients. Average duration from the first intake of substance use in substance-dependent patient group was 10.24 (±7.008) years.

### 3.2. Descriptive Variables

Schizophrenia patients with SUD were significantly older than their non-substance-using counterparts (Table 1). They were predominantly male (*x* ^2^ = 11.45; significance = 0.001). There was no significant difference in education, total duration of illness, and age of onset, although the duration of untreated illness was significantly longer in patients with substance use disorder (Table 1). There were no significant differences in occupation (*x* ^2^ = 1.42; significance = 0.701), marital status (*x* ^2^ = 2.91; significance = 0.088), family type (*x* ^2^ = 1.74; significance = 0.187), and residence (*x* ^2^ = 0.022; significance = 0.882). Total per day antipsychotic dose (chlorpromazine equivalent in mg) used was similar between groups (Group (1): 350.28 ± 122.45; Group (2): 356.80 ± 146.59;* P* = 0.773) (Table 1). Both groups had similar history of suicidal attempts (*x* ^2^ = 0.032; significance = 0.858). Number of suicidal attempts also did not show any differences (Table 1).

### 3.3. Psychopathology at Presentation

At presentation there were no differences in positive, negative, or general psychopathology scale of PANSS and variables in HARS. There was also no difference in variables of BPRS except mannerism. Mannerism was higher in patients who did not use any substance (Table 2). However, persons with substance use disorder had significantly more depression as evident by total Calgary score (Table 2). They also had significantly more hopelessness, self-depreciation, suicidal ideation, and observed depression (Table 2). The substance use disorder group also tended to have earlier waking. However, there were no differences in depressed mood, guilty ideas of reference, pathological guilt, and morning depression (Table 2).

### 3.4. Psychopathology at Three Weeks

Schizophrenia patients with substance use disorder had poorer abstraction. There were no other differences between the groups after three weeks in the ratings of HARS, BPRS, and positive, negative, or general psychopathology scale of PANSS (Table 3). However, schizophrenia patients with substance use disorder had significantly more depression, self-depreciation, and observed depression compared to schizophrenia patients without history of substance use. The first group also tended to have higher hopelessness and total Calgary score (Table 3).

### 3.5. Psychopathology at Six Weeks

Patients with substance use disorder continued to have more difficulty in abstraction and earlier waking, self-depreciation, and a trend towards more depressed mood and total Calgary score. Similarly, patients without substance use tended to have more disturbed volition (Table 4). There were no differences in any of the variables of HARS, BPRS, Calgary scale for depression, and positive, negative, or general psychopathology scale of PANSS (Table 4).

### 3.6. Side Effects

There were no differences between the groups in side effects at three weeks (Group (1) 7.18 ± 4.42; Group (2) 7.20 ± 4.55;* t* = −0.019; sig = 0.985) or at six weeks (Group (1) 4.96 ± 4.28; Group (2) 4.42 ± 3.96;* t* = 0.719; sig = 0.474).

### 3.7. Symptom Resolution

To examine whether there was any difference between the rate of symptom resolution between schizophrenia patients with substance use disorder and without substance use disorder, we applied a general linear model repeat measure ANOVA. Greenhouse-Geisser correction was used as Mauchly's test of sphericity revealed a significance level less than 0.05. No difference in symptom resolution was observed between groups (Table 5).

## 4. Discussion

Majority of participants were male which was probably due to infrequent admission of females in inpatient psychiatric facilities in India. 51% of participants were married, which was higher compared to a 30% marriage rate reported by Bhatia et al. [33], who also demonstrated that, in US sample, majority of patients had a single status in comparison to Indian patients. The difference in marital status may be due to a predominantly rural population in our sample (*n* = 109; 75.2%) compared to a predominantly urban sample in the study by Bhatia et al. [33]. In rural Indian culture, marriage is a common social norm which is compounded by a popular belief of marriage curing mental illnesses. Majority of our participants were living in extended families, which may indicate the acceptance of schizophrenia in Indian society. As having a spouse and living with families give more social support than living alone, this may explain why prognosis of schizophrenia is better in India compared to many Western countries [19].

### 4.1. Prevalence of Substance Use

Prevalence of substance use disorders in our sample was 63.6% which is similar to that reported in other recent studies [34–36]. Nicotine was the commonest substance abused which is supported by other studies [37]. Cannabis was the second most common substance used followed by alcohol. The prevalence of substance use disorders in schizophrenia patients in the present study is similar to the rates shown in another Indian study [38].

Interestingly, an earlier Indian study reported an even lower rate of substance use (38%) in schizophrenia patients [39] though this study was carried out at a different geographical location on patients from different cultural background. The lower prevalence of nicotine smoking reported in this study might also be due to the fact that outpatients were evaluated. Since we evaluated inpatient population, we may have been dealing with more severe cases where high prevalence of substance abuse is expected. Another possibility also might be there. Our sample population was mainly rural in their residential background. Cuffel [40] argued that dual diagnosis was commoner in rural population because of lack of necessary facilities to deal with the problem of substance abuse.

The overall lower prevalence of substance abuse reported in Indian research (including our study) compared to the western literature may be due to differences in the availability of substances and socioeconomic background. The reasons why psychiatric patients in Indian studies did not smoke more could be predominantly socioeconomic and cultural. Most of them were unemployed and poor and had no independent source of income. The patients were dependent on the family to give money to buy cigarettes or “bidis.” The family members could exercise some control over the smoking habit of the patients as all the patients in the present study were living with their families. Moreover, in India the patients do not receive any disability assistance from the state that could be spent on smoking as it happens in the developed countries where such assistance is available. The economic factor along with the cultural restrictions mentioned earlier could have curbed smoking behavior by the patients [39].

Till now all epidemiological data on dual diagnosis were based on diagnoses of substance abuse and dependence. However, the new DSM-5 diagnostic criteria combine abuse and dependence criteria into one disorder, with two additional changes (exclusion of legal problem criterion and inclusion of craving criterion) [41]. It is therefore likely that there will be an increase in dual diagnoses in the future.

### 4.2. Demographic Variables

There was no difference in the age of onset between persons with and without substance use disorders. Interestingly, the duration of illness before coming to the attention of psychiatric services was longer in patients with substance abuse. This may have two possibilities. First, substance-using patients might be more intact than non-substance-using patients. So they required to be brought to a psychiatric facility later than substance nonusers. Second, the persons had more opportunity to pick up the habit of using substances as they were brought to the attention of specialized psychiatric services later. Hence, instead of being a marker of better intactness, substance use may simply be a result of delayed treatment.

There was a significant gender difference in the patterns of substance use. Although there was inadequate representation of females in the study population, the gender difference in substance may indicate a role of cultural factors on substance use pattern as substance use in females is uncommon in India [42].

There were no differences in schizophrenia patients with and without substance use disorder with regard to education, occupation, family type, residence, marital status, per day total antipsychotic dose, or past suicidal attempt. This may be surprising as it implies that dual diagnosis patients were at least equally intact as single diagnosis patients.

Indeed, the extent to which substance use disorder alters the clinical course of schizophrenia in a more chronic direction is still debated in the world literature. Not all studies indicate that the prognosis of dual-diagnosed patients is necessarily poor. A number of studies have found no relationship between substance use disorder and symptomatology [43]. Zisook et al. [44] found no differences in the percentages of being married, living alone, receiving disability, work status, mean number of length, or medication doses. These findings, together with reports of better premorbid adjustment, less overall psychopathology [3], fewer brain structural abnormalities, and fewer hospitalizations [43] might indicate that patients prone to substance use disorder may be more socially competent and thus less severely ill.

### 4.3. Symptom Variables

This study did not reveal any overall difference between SUD and nonuser schizophrenic patients in positive symptoms, negative symptoms, general psychopathology symptoms, or anxiety. However, there was significantly higher depression as well as several depressive symptoms reported in patients with SUD at presentation, at three weeks, and at six weeks. There might be two possible explanations for these observations. Firstly, substance use disorder could have produced symptoms of depression in users which persisted throughout the study period of six weeks, or alternatively patients who had more symptoms of depression and tried to self-medicate them with substances. There was visible decrease in depressive symptoms from baseline to six weeks. However, whether this is because of abstinence or treatment cannot be confirmed.

Patients with substance use disorder revealed more difficulty in abstraction after three weeks and six weeks, but this difference was surprisingly absent at presentation. Logically this may imply uncovering of abstraction difficulty after forced abstinence in the substance abusing group. In other words, substance abuse might have helped to mask difficulty in abstraction during presentation. Interestingly nicotine was the commonest abusing substance, which has been postulated to activate central nicotinergic receptors and improving cognitive functions [45].

Whether these findings imply a better adjustment in substance using schizophrenia patients will be an interesting area of further research. Indeed, Dixon et al. [3] put forward an interesting hypothesis. According to them, social skills and motivation necessary to obtain substances might select out in the dual-diagnosis patients a subgroup of schizophrenia patients with better prognosis and overall outcome.

“Self-medication hypothesis” of substance abuse in schizophrenia suggests that schizophrenic patients use substances to self-medicate either depression [46], positive or negative symptoms [47], or extrapyramidal symptoms [9]. Our present study favored the possibilities that patients with schizophrenia used substances to self-medicate depression as well as negative symptoms like difficulty in abstraction, but not psychotic symptoms or extrapyramidal symptoms.

There have been several studies which have supported such a hypothesis. Noordsy et al. [48] reported that alcohol relieved a variety of unpleasant nonpsychotic symptoms rather than the positive symptoms of psychosis. In their study, alcohol often relieved the dysphoria rather than the psychosis itself. In another study, Addington and Duchak [7] observed that dual-diagnosed patients used substances to self-medicate most commonly to relieve depression and improve affect. Hamera et al. [49] showed that intake of caffeine increased in schizophrenia patients whenever they felt tensed and depressed. In another study, all patients reported that substances acutely increased happiness and decreased depression. Most patients took substances to relieve depression and relax [3].

There was no difference in our study between schizophrenia patients with SUD and nonusers regarding symptom remission. This may indicate that SUD did not modify the course of schizophrenic illness, treatment response, and recovery, at least in the short term which is similar to the findings of Dixon et al. [3]. There were no differences in the total doses of antipsychotic required to achieve remission in both groups, which is similar to the findings of an earlier study [3]. Patkar et al. [50] also found no differences in typical and atypical antipsychotic dosages in smoking and nonsmoking schizophrenic patients. There were also no differences in side effects, particularly extrapyramidal side effects, between the groups. This may indicate that mitigating extrapyramidal side effects was not a cause for taking substances. Similar dosage requirement and side effect profile in both groups may indicate similar treatment response in schizophrenia patients in both groups at least in short term when substance abuse was controlled. This may bear a significant message for the treatment of schizophrenia. If substance use disorder is controlled, the outcome of dual-diagnosis patients becomes no different from single-diagnosis schizophrenia patients.

## 5. Limitations

Limitations of the study include possible selection bias being a tertiary care hospital based study where patients with more severe illness are more likely to be included. The lack of sample size statistical calculation, randomization procedures to reduce the potential selection bias, and statistical methods to control for potential confounders are limitations. No structured diagnostic interviews (e.g., SCID-I) were used to assess schizophrenia and alcohol/substance use disorder. No tests were carried out to confirm substance use, and diagnosis of substance use was based on statement of patients and their relatives.

## 6. Conclusion

Comorbid SUD was detected in 63.6% of 143 consecutive schizophrenia patients from Eastern India. Nicotine was the commonest substance followed by cannabis and alcohol. Schizophrenia patients with SUDs had longer duration of untreated illness and more depressive symptoms at presentation and six-week follow-up. Schizophrenia patients with SUD should be carefully assessed for presence of depression.

## Figures and Tables

**Table 1:** Sociodemographic variables of schizophrenia patients with substance use disorder (Group 1) and without substance use disorder (Group 2).

Variables	Group 1 (*n* = 91) Mean ± s.d.	Group 2 (*n* = 52) Mean ± s.d.	*t*	Sig	CI Lower bound	CI Upper bound
Age in years	31.01 ± 8.16	27.84 ± 7.41	2.305	0.023	0.450	5.88
Education years	9.31 ± 4.66	9.90 ± 4.40	−0.736	0.463	−2.156	0.99
Total duration of illness in days	2265 ± 1982	1772 ± 1462	1.563	0.120	−130.40	1115.10
Duration of untreated illness in days	1891 ± 1730	1302 ± 1149	2.190	0.030	57.246	1119.81
Age of onset in years	25.08 ± 7.95	23.15 ± 6.49	1.492	0.138	−0.628	4.49
CPZ equivalent antipsychotic dose in mg/day	350.28 ± 122.45	356.80 ± 146.59	−0.289	0.773	−57.720	33.81
Number of suicidal attempts	0.17 ± 0.46	0.17 ± 0.46	0.035	0.972	−0.1521	0.157

CI: confidence interval; “*t*”: *t* statistic; degree of freedom = 141.

**Table 2:** PANSS, HARS, and BPRS and Calgary scale for depression scores at baseline in schizophrenia patients with substance use disorder (Group 1) and without substance use disorder (Group 2).

Scales	Subscales	Group 1 (*n* = 91) Mean ± s.d.	Group 2 (*n* = 52) Mean ± s.d.	*t*	Sig	CI Lower bound	CI Upper bound
PANSS	Positive scale	23.83 ± 6.41	23.94 ± 6.19	−0.097	0.923	−2.284	2.070
	Negative scale	26.21 ± 8.05	26.46 ± 7.41	−0.178	0.859	−2.931	2.447
	Negative scale Difficulty in abstraction	3.74 ± 1.25	3.32 ± 1.58	1.71	0.089	−0.063	0.882
	General psychopathology scale	44.01 ± 7.19	45.23 ± 7.44	−0.963	0.337	−3.724	1.284

HARS	Total HARS score	17.90 ± 8.74	15.71 ± 7.48	1.515	0.132	−0.668	5.047

BPRS	Total BPRS score	33.93 ± 8.06	34.71 ± 7.41	−0.571	0.569	−3.470	1.915
	Mannerism/posturing	0.32 ± 0.74	0.67 ± 1.06	−2.262	0.025	−0.643	−4.33

Calgary scale for depression	Depression	1.35 ± 1.13	1.09 ± 0.93	1.374	0.172	−0.112	0.623
	Hopelessness	0.95 ± 1.09	0.44 ± 0.75	3.002	0.003	0.175	0.852
	Self-depreciation	0.86 ± 1.02	0.51 ± 0.85	2.079	0.039	1.714	0.680
	Guilty ideas of reference	0.45 ± 0.91	0.21 ± 0.66	1.656	0.100	−4.632	0.524
	Pathological guilt	0.37 ± 0.82	0.25 ± 0.62	0.938	0.350	−0.136	0.384
	Morning depression	0.68 ± 0.98	0.42 ± 0.80	1.607	0.110	−5.942	0.575
	Early waking	0.90 ± 1.14	0.55 ± 0.84	1.884	0.062	−1.682	0.703
	Suicide	0.76 ± 1.13	0.19 ± 0.48	3.480	0.001	0.249	0.904
	Observed depression	1.21 ± 1.18	0.75 ± 0.94	2.451	0.015	9.092	0.848
	Total Calgary score	7.57 ± 7.77	4.44 ± 5.63	2.543	0.012	0.696	5.561


CI: confidence interval; “*t*”: *t* statistic; degree of freedom = 141.

**Table 3:** PANSS, HARS, BPRS, and Calgary scale for depression scores at 3 weeks in schizophrenia patients with substance use disorder (Group 1) and without substance use disorder (Group 2).

	Variables	Group 1 (*n* = 91) Mean ± s.d.	Group 2 (*n* = 52) Mean ± s.d.	*t*	Sig	CI Lower bound	CI Upper bound
PANSS	Positive scale	17.00 ± 6.80	16.71 ± 7.63	0.23	0.81	−2.15	2.73
	Negative scale Total score	19.52 ± 7.97	19.07 ± 7.25	0.33	0.73	−2.20	3.10
	Negative scale Difficulty in abstraction	3.64 ± 1.53	3.03 ± 1.65	2.21	0.02	0.66	1.15
	General psychopathology scale	31.61 ± 7.86	32.26 ± 8.67	−0.460	0.64	−3.461	2.153

HARS	Total score	10.43 ± 7.30	9.15 ± 6.33	1.06	0.29	−1.11	3.68

BPRS	Total score	19.85 ± 9.77	19.53 ± 10.49	0.18	0.85	−3.13	3.76
	Mannerism/posturing	0.27 ± 0.65	0.46 ± 0.61	−1.689	0.09	−0.41	−0.40

Calgary scale for depression	Depression	0.85 ± 0.91	0.50 ± .72	2.41	0.01	6.46	0.64
	Hopelessness	0.43 ± 0.77	0.23 ± .54	1.70	0.09	−3.27	0.450
	Self-depreciation	0.42 ± 0.74	0.15 ± .41	2.44	0.01	5.23	0.49
	Guilty ideas of reference	0.20 ± 0.56	0.13 ± .52	0.77	0.44	−0.11	0.26
	Pathological guilt	0.16 ± 0.50	0.16 ± .35	0.87	0.38	−8.73	0.22
	Morning depression	0.27 ± 0.59	0.21 ± .53	0.63	0.52	−0.13	0.26
	Early waking	0.39 ± 0.77	0.28 ± .63	0.84	0.39	−0.14	0.35
	Suicide	0.25 ± 0.69	0.13 ± .39	1.12	0.26	−8.90	0.32
	Observed depression	0.68 ± 0.89	0.38 ± .77	2.00	0.04	4.31	0.58
	Total Calgary score	3.70 ± 5.02	2.13 ± 3.84	1.94	0.05	−2.47	3.16

CI: confidence interval; “*t*”: *t* statistic; degree of freedom = 141.

**Table 4:** PANSS, Calgary scale for depression, HARS, and BPRS scores at 6 weeks in schizophrenia patients with substance use disorder (Group 1) and without substance use disorder (Group 2).

	Variables	Group 1 (*n* = 91) Mean ± s.d.	Group 2 (*n* = 52) Mean ± s.d.	*t*	Sig	CI Lower bound	CI Upper bound
PANSS	Positive scale	13.09 ± 5.84	13.23 ± 5.86	−0.130	0.897	−2.143	1.879
	Negative scale	16.10 ± 7.63	14.98 ± 6.96	0.878	0.382	−1.413	3.672
	Negative scale Difficulty in abstraction	3.26 ± 1.59	2.67 ± 1.65	2.100	0.038	0.03	1.146
	General scale	25.84 ± 6.72	26.61 ± 7.63	−0.626	0.532	−3.198	1.659

HARS	Total score	6.23 ± 6.89	4.84 ± 5.01	1.269	0.207	−0.773	3.548

BPRS	Total score	12.45 ± 9.11	12.17 ± 8.95	0.176	0.860	−2.835	3.390
	Mannerism/posturing	0.23 ± 0.45	0.35 ± 0.48	−1.441	0.152	−0.27	−0.42

Calgary scale for depression	Depression	0.50 ± 0.77	0.28 ± 0.63	1.707	0.090	−0.03	0.468
	Self-depreciation	0.29 ± 0.67	0.09 ± 0.35	1.987	0.049	0.001	0.400
	Early waking	0.24 ± 0.63	0.03 ± 0.19	2.235	0.027	0.02	0.383
	Total score	2.24 ± 4.58	0.98 ± 2.53	1.827	0.070	−0.103	2.625

CI: confidence interval; “*t*”: *t* statistic; degree of freedom = 141.

**Table 5:** Resolution of symptom in schizophrenia patients with substance abuse (Group 1, *n* = 91) and without substance abuse (Group 2, *n* = 52): general linear model repeat measure ANOVA.

Variable	Group	Baseline	After 3 weeks	After 6 weeks	*F*	*P*
PANSS positive scale	Group 1	23.83 ± 6.41	17.00 ± 6.80	13.09 ± 5.84	0.766	0.454
	Group 2	23.94 ± 6.19	16.71 ± 7.63	13.23 ± 5.86		

PANSS negative scale	Group 1	26.21 ± 8.05	19.52 ± 7.97	16.10 ± 7.63	1.090	0.325
	Group 2	26.46 ± 7.41	19.07 ± 7.25	14.98 ± 6.96		

PANSS general scale	Group 1	44.01 ± 7.19	31.61 ± 7.86	25.84 ± 6.72	0.119	0.865
	Group 2	45.23 ± 7.44	32.26 ± 8.67	26.61 ± 7.63		

PANSS total	Group 1	93.95 ± 14.10	67.89 ± 17.95	54.82 ± 16.65	0.157	0.832
	Group 2	95.55 ± 12.05	68.40 ± 18.07	55.16 ± 16.24		

Calgary	Group 1	7.57 ± 7.77	3.70 ± 5.02	2.24 ± 4.58	1.391	0.249
	Group 2	4.44 ± 5.63	2.13 ± 3.84	0.98 ± 2.53		

HARS	Group 1	17.90 ± 8.74	10.43 ± 7.30	6.23 ± 6.89	0.013	0.980
	Group 2	15.71 ± 7.48	9.15 ± 6.33	4.84 ± 5.01		

BPRS	Group 1	33.93 ± 8.06	19.85 ± 9.77	12.45 ± 9.11	0.155	0.832
	Group 2	34.71 ± 7.41	19.53 ± 10.49	12.17 ± 8.95		

Mauchly's test of sphericity sig.: 0.000; *F*: Greenhouse-Geisser correction.
